# Metavirome Identification and Pathogenicity Evaluation of Tibet Orbivirus in Pigs

**DOI:** 10.1155/tbed/6628384

**Published:** 2025-11-17

**Authors:** Zhanhong Li, Pei Zhu, Zhenxing Zhang, Zhuoran Li, Peng Liu, Li Meng, Qiuyan Yang, Zhen Yang, Jianling Song

**Affiliations:** ^1^Yunnan Tropical and Subtropical Animal Virus Diseases Laboratory, Yunnan Animal Science and Veterinary Institute, Kunming, China; ^2^Key Laboratory of Transboundary Animal Diseases Prevention and Control (Co-construction by Ministry and Province), Ministry of Agriculture and Rural Affairs, Kunming, China; ^3^Mile Preventive and Control Center for Animal Diseases, Taoyuan Road, Mile, China; ^4^Honghe Preventive and Control Center for Animal Diseases, Zhongzhao Road, Mengzi, China

**Keywords:** pathogenicity evaluation, piglets, Tibet orbivirus, viral metagenomic sequencing

## Abstract

Tibet orbivirus (TIBOV) is an orbivirus transmitted by mosquitoes and *Culicoides*, despite specific neutralizing antibodies being detected in pigs, but the molecular genetic characteristics of TIBOV strains in infected pigs are completely uncharted, and their pathogenicity in piglets is poorly elucidated. This study aimed to investigate the genetic characteristics of TIBOV in infected pigs and evaluate the pathogenicity of TIBOV in weaned piglets. Through viral metagenomic sequencing, seven segments (VP1-VP4, VP6, NS1, and NS2) of TIBOV were obtained from swine tissues, and the sequences showed high identity with TIBOVs isolated from *Culicoides*, mosquitos, and cattle. After infection with TIBOV, the body temperature, appetite, and behavior of the piglets were normal, whereas hemorrhage nodes were observed on the hooves of all piglets and on the abdominal skin of one pig. Viremia was first detected at 2 days postinfection (dpi), peaked at 6 dpi, and remained high until 21 dpi. The virus was distributed in multiple organs, and the highest viral load and strongest viral nucleic acid signals were observed in the spleen. The most severe lesion was observed in the spleen with white pulp atrophy, a decreased number of lymphocytes, and widened septa of the medullary cord, indicating that the spleen was the most important target organ of TIBOV infection. The levels of inflammatory cytokines, including interleukin (IL)-18, tumor necrosis factor-α (TNF-α), interferon (IFN)-α, and IFN-λ3 in peripheral blood lymphocytes decreased significantly from 2 to 6 dpi, and interferon-stimulated gene-15 (*ISG-15*) and IFN regulatory factor 7 (*IRF-7*) expression levels declined significantly from 2 to 9 dpi, suggesting that the host immune response was inhibited within 6 dpi. Our findings confirmed that TIBOV elicited long-term viremia with mild clinical symptoms in piglets, the spleen was the target organ of TIBOV proliferation, and the host immune response may be slightly inhibited in the early stage of viral infection.

## 1. Introduction

Tibet orbivirus (TIBOV) is a member of the genus *Orbivirus* within the family *Reoviridae* [1]. The genome of the TIBOV consists of 10 double-stranded RNA (dsRNA) segments (Seg 1–10), and the nucleotide sequence length of each segment ranges from 3950 bp (Seg 1) to 832 bp (Seg 10) [2]. All segments show the same structure containing a 5′ untranslated region (UTR), a coding sequence (CDS), and a 3′ UTR, and they encode three nonstructural proteins (NS1–3) and seven structural proteins (VP1–7) [3]. The viral capsid consists of outer, intermediate, and inner capsid proteins. Similar to the homogenous proteins of other orbiviruses (e.g., bluetongue virus [BTV] and epizootic hemorrhagic disease virus [EHDV]), the outer capsid protein VP2 of TIBOV is highly variable. TIBOV strains can be divided into six serotypes (TIBOV-1 to TIBOV-6) based on the diversity of the VP2 protein. TIBOV-1, -2, -5, and -6 were isolated from China, and TIBOV-4 and -5 were isolated from Japan [4].

The original strain of TIBOV (XZ0906) was isolated from mosquitoes (*Anopheles maculatus*) collected in Motuo County, Tibet, China, in 2009 [1]. Subsequently, several TIBOV strains were isolated from *Culicoides* (DH13C120, YN12246, YNV/KM-1, YNV/17-14, V290/YNSZ), mosquitoes (D181/2008), and cattle blood (V298/YNJH) collected in China [4–9] and two TIBOV strains were isolated from *Culicoides* (KSB-8/C/09, KSB-3/C/10) in Japan [2]. Furthermore, TIBOV strain P110 (GenBank accession: MH267259–MH267268) was isolated from mosquitoes in Nepal. In summary, all TIBOV strains were isolated from Asia. Specific antibodies against TIBOV were detected in livestock serum specimens collected in Yunnan Province with the following positivity rates: cattle (44%), buffaloes (20%), goats (4%), and pigs (14%) [6, 8]. Antibodies were detected through plaque-reduction neutralization tests [8] or indirect immunofluorescence assays [4], and both tests use special TIBOV isolates. However, different TIBOV serotype strains prevalent in animals have been detected through serotype-specific quantitative reverse transcription polymerase chain reaction (qRT-PCR) [4], so we infer that the serological investigations in previous studies did not cover all serotype strains circulating in animals, and the actual serum positivity rate was possibly higher than that reported previously.

TIBOV proliferates efficiently in cell lines derived from mosquitoes, monkeys, pigs, humans, and hamsters and can cause serious illness and mortality in lactating mice through experimental infection [4, 8]. Aggregating the results of epidemiological (serological investigations and molecular detection), host cell tropism, and mouse infection experiments, TIBOV shows potential to infect different domestic animals, but its pathogenicity in animals is completely unknown. In this study, the pathogenicity of TIBOV in piglets was assessed. Our findings expand our knowledge of the pathogenicity of this virus.

## 2. Materials and Methods

### 2.1. Tissue Sample Collection

Twenty-one dead pigs from commercial pig farms were dissected, and the spleen, nodus lymphaticus mandibularis, and inguinal lymph nodes were collected from a Biosafety Disposal Center in Mile County of Yunnan Province in October and December 2023. The collected tissues were separated into six groups based on the collection date and location (Table 1). All tissues were stored at ‒80°C until sequencing.

### 2.2. Nucleic Acid Extraction and Reverse Transcription

Each of the spleen, nodus lymphaticus mandibularis, and inguinal lymph node tissues (0.1 g) from each group were mixed and homogenized with stainless steel beads of 2 mm in diameter (EASYBIO, Beijing, China) in 1 mL minimal essential medium (MEM) using a TissueLyser II homogenizer (Qiagen, Hilden, Germany). The samples were then centrifuged at 8000 × *g* for 15 min at 4 °C to remove the cell debris, and the resulting supernatant was filtered through a 0.45-µm-pore filter (Sartorius, Göttingen, Germany). To eliminate host genomic DNA and dissociative nucleic acid in the samples, 25 U Benzonase nuclease (Merck Millipore, Darmstadt, Germany), 14 U of Turbo DNase I (Thermo Fisher Scientific, Waltham, MA, USA), and 20 U of RNase A (Sangon Biotech, Shanghai, China) were added to 127 µL of the supernatants to a final volume of 150 µL. The samples were then digested at 37°C for 1 h, then incubated at 65°C for 10 min to inactivate the nuclease. Total viral nucleic acids in the obtained products were extracted using a QIAamp MinElute Virus Spin Kit (Qiagen) according to the manufacturer's instructions. The extracted viral RNA was quantified using the Equalbit RNA HS Assay Kit (Vazyme Biotech Co., Ltd., Nanjing, China). Viral RNA was reverse-transcribed with the primer K-random-s (5′-GACCATCTAGCGACCTCCACNNNNNNNN-3′) and Superscript III reverse transcriptase (Thermo Fisher Scientific), after which the Klenow fragment polymerase (New England Biolabs, Ipswich, MA, USA) was added to synthesize double-stranded cDNA. Subsequently, sequence-independent single-primer amplification was performed to obtain sufficient viral nucleic acids using AccuPrime Taq DNA Polymerase (Invitrogen, Carlsbad, CA, USA) according to the manufacturer's protocol. PCR products were purified using Agencourt AMPure Beads (Beckman-Coulter, Villepinte, France) according to the manufacturer's instructions.

### 2.3. Next-Generation Sequencing

cDNA libraries were constructed using the VAHTS Universal Plus DNA Library Prep Kit for Illumina (Vazyme Biotech Co., Ltd.). Briefly, the purified PCR products were ultrasonicated, and dATP and Klenow fragment were added to produce 3′-dA overhangs. The DNA fragments were then ligated to Illumina adaptors and amplified using PCR with adaptor-specific primers. The constructed cDNA libraries were validated using an Agilent 4200 Bioanalyzer (Agilent Technologies, Santa Clara, CA, USA) and quantified using a Qubit2.0 fluorometer (Life Technologies, Waltham, MA, USA). Virome sequencing was conducted at Chengdu Phagetimes Biotech Co., Ltd. (Chengdu, China) using a NovaSeq 6000 platform (Illumina, San Diego, CA, USA). The quality of the raw reads was assessed using FastQC version 0.12.0 (https://www.bioinformatics.babraham.ac.uk/projects/fastqc/); adapter removal, decontamination, and error correction of the raw reads were performed using BBDuk version 37.90 (https://jgi.doe.gov/data-and-tools/bbtools/bb-tools-user-guide/bbduk-guide/, accessed on 20 July 2020); and host sequences were removed using Bowtie2 (v2.3.4.3) [10]. After filtration and trimming, de novo assembly was performed using metaSPAdes. Longer contigs were defined as significant data, and open reading frames were predicted using prodigal (v 2.6.3) [11].

### 2.4. Computational and Phylogenetic Analysis

To identify the viral sequences, the assembled contigs were compared to the National Center for Biotechnology Information (NCBI) protein database (nonredundant [NR] proteins) using Diamond [12] and the NCBI nucleotide database using the BlastN algorithm with an *E*-value < 10^5^. The top-hit contigs were confirmed as known viral sequences. The mismatching contigs (average nucleotide identity <80% and query coverage <50%) were analyzed using VirBot [13] and VirSorter2 [14] to predict the unknown virus sequences and were annotated using VPF-Class [15]. The confirmed TIBOV contigs were aligned with the sequences of other reference TIBOV strains from the GenBank database using MEGA 6.06 software [16]. Phylogenetic trees were generated using the neighbor-joining method in MEGA 6.0, and 1000 bootstrap replicates were used to evaluate the reliability of the phylogenetic trees.

### 2.5. Virus Preparation for Animal Experiments

TIOBV strain V290/YNSZ (GenBank accession numbers: ON040940 to ON040949) used in this study was isolated from *Culicoides* collected from Shizong County of Yunnan Province in 2022, as documented in our previous study [4], and stored at ‒80°C. *Aedes albopictus* C6/36 cells (the China Center for Type Culture Collection, Wuhan, China) were cultured in 75 cm^2^ flasks at 28°C in a 5% CO_2_ incubator with 45% Dulbecco's modified Eagle's medium (DMEM) and 45% Roswell Park Memorial Institute 1640 medium (Invitrogen, CA, USA) containing 10% heat-inactivated fetal bovine serum, 0.1 mg/mL streptomycin, and 100 U/mL penicillin. The virus stocks were thawed at room temperature, inoculated into C6/36 cell monolayers, and cultured for 4 days until a characteristic cytopathic effect (CPE) was observed. The cells and culture medium were then harvested, subjected to three freeze–thaw cycles, and centrifuged at 8000 × *g* for 15 min at 4°C to remove the cell pellets. The tissue culture infection dose (TCID_50_) of the virus was determined using the Reed–Muench method [17].

### 2.6. Experimental Infection of Piglets

Seven 28-day-old piglets were purchased from a commercial piggery company in Yunnan Province, China, and all pigs were confirmed to be negative for TIBOV, porcine reproductive and respiratory syndrome virus (PRRSV), classical swine fever virus (CSFV), porcine circovirus (PCV), porcine parvovirus (PPV), pseudorabies virus (PRV), porcine epidemic diarrhea virus (PEDV), swine transmissible gastroenteritis virus (TGEV), and porcine deltacoronavirus (PDCoV) using virus-specific RT-PCR methods [4, 18–20]. The animals were randomly divided into two groups (Group A, *n* = 4 and Group B, *n* = 3) and housed in separate barns with free access to food and drinking water. After 3 days of adaptation, piglets in the challenged group (Group A) were intravenously inoculated with the V290/YNSZ strain (5.0 × 10^6^ TCID_50_/mL, 3 mL/head), and those in Group B received 3 mL of C6/36 cell culture medium supernatant as a negative control. The mental status, appetite, body weight, rectal temperature, respiratory symptoms, fecal status, hair status, and skin lesions of all piglets were monitored and recorded daily. Blood samples were collected from all animals at 0, 2, 4, 6, 9, 12, 15, 18, and 21 dpi to determine the viral load and cytokine mRNA levels. Serum was collected simultaneously to determine the titers of neutralizing antibodies (nAbs) against V290/YNSZ. All piglets were euthanized at 21 dpi, and gross lesions in the organs were observed through necropsy. The heart, lung, liver, spleen, and brain were then collected. Half of the tissue samples was placed in a 5 mL sterile tube and stored at −80°C for future testing, while the other half was fixed with 4% neutral-buffered formaldehyde (Servicebio, Wuhan, China) for further histological evaluation.

### 2.7. Viremia, and Tissue Virus Load Detection

To monitor viremia and the viral load in different tissues after virus challenge, 0.5 g of the tissues was homogenized in 1 mL of sterile phosphate-buffered saline (PBS) using a TissueLyser II homogenizer (Qiagen) and centrifuged at 8000 × *g* for 10 min at 4 °C. The viral RNA was then extracted from 50 μL of the homogenized tissue supernatant and EDTA anticoagulant blood with the MagMAX magnetic beads viral RNA isolation kit on the KingFisher Flex platform (Applied Biosystem, Foster, CA, USA). The number of viral copies was determined using the TIBOV pan-RT-qPCR method developed in our previous study [4].

### 2.8. Serum nAb Detection

The titer of nAbs against V290/YNSZ was determined using a serum neutralization test (SNT), as previously described [17]. First, the serum samples were incubated at 56°C for 30 min to inactivate the complements. The samples were then two-fold serially diluted with DMEM (1:4 to 1:1024). Subsequently, the V290/YNSZ stock (5.0 × 10^6^ TCID_50_/mL) was diluted to 100 TCID_50_/50 µL, and 50 µL of the diluted virus was added to an equal volume of serum in 96-well-plates in triplicate. The samples were incubated at 37°C for 1 h, after which 50 µL aliquots of C6/36 cells (~2.5 × 10^4^ cells per well) were seeded into all of the wells, and they were cultured at 28°C with 5% CO_2_ for 4 days. The CPEs were then recorded, and the nAb titer of the serum was defined as the highest serum dilution that inhibited 50% of viral growth compared to the negative serum control.

### 2.9. Cytokine mRNA Levels and Concentrations Detection

Total RNA was extracted from 500 µL aliquots of whole blood samples using the RNAprep Pure Hi-Blood Kit (TIANGEN Bio, Beijing, China). 1 μg of RNA was converted to cDNA using the PrimeScript RT reagent kit with gDNA eraser (Takara Bio, Dalian, China) following the manufacturer's instructions. cDNA was used to evaluate the gene expression levels of interleukin (IL)-1β [21], *IL-6* [21], *IL-18* [22], tumor necrosis factor-α (*TNF-α*) [21], interferon (IFN)-α [23], *IFN-β* [24], *IFN*-*λ3* [24], interferon-stimulated gene 15 (*ISG-15*) [25], and IFN regulatory factor 7 (*IRF-7*) [26] using qPCR with previously described primer sets (Table S1), and β-actin [23] was used as an endogenous control to normalize the amount of input cDNA. qPCR reactions were performed using TB Green Fast qPCR Mix Kit (Takara Bio) and a 7500 Fast Real-Time PCR System (Applied Biosystems, Thermo Fisher Scientific), according to the manufacturer's recommendations. The relative quantification of mRNA expression levels of the target genes was calculated using the 2^−ΔΔCt^ method according to a previous report [27], and the results are presented as the fold change relative to the levels in control animals.

Meanwhile, the concentrations of IL-1β, IL-6, IL-18, TNF-α, IFN-α, IFN-β, and IFN-λ3 in the serum samples were measured using commercial ELISA kits (Meimian, Yancheng, China) and following the manufacturer's instructions.

### 2.10. Histopathological Examination

The heart, liver, spleen, lungs, and brain were fixed with 4% neutral-buffered formaldehyde and stained with hematoxylin and eosin, as described previously [28], to evaluate histological lesions. To detect the distribution of TIBOV nucleic acids in the heart, liver, spleen, lungs, and brain, an RNA-based tyramide signal amplification fluorescence in situ hybridization (TSA-FISH) assay was performed as previously reported [28]. Briefly, the TIBOV-P RNA probe targeting the *VP7* gene of V290/YNSZ (position: 48–347) was labeled with digoxigenin (Roche, Mannheim, Germany). The fixed tissues were dehydrated in an alcohol gradient (Sinopharm Chemical Reagent Co., Ltd., Shanghai, China), embedded in paraffin (Sakura, Tokyo, Japan), and sectioned using a microtome (Shanghai Lycra, Shanghai, China). The sections were deparaffinized in xylene (Sinopharm Chemical Reagent Co., Ltd.) and rehydrated through a gradient of alcohol washes, followed by treatment with 3% hydrogen peroxide (Sinopharm Chemical Reagent Co., Ltd.) in PBS for 20 min. The sections were boiled in citric buffer for 15 min and incubated with 20 μg/mL proteinase K (Servicebio) for 30 min at 37°C. The sections were incubated with 3% methanol-H_2_O_2_ (Servicebio) to block endogenous peroxidase, prehybridized with hybridization buffer (50% formamide [v/v], 900 mM NaCl, 20 mM Tris-HCl, pH 7.2) (Servicebio) at 37°C for 1 h, and subsequently hybridized with the TIBOV-P probe (500 nM) in hybridization buffer overnight at 40°C. After serial washes with saline sodium citrate (SSC) buffer (Servicebio) at different concentrations (2× to 0.5×), the sections were blocked with 10% (v/v) rabbit serum (Servicebio) at room temperature for 30 min. Mouse anti-digoxigenin-labeled peroxidase (Roche) was added, and the samples were incubated at 37°C for 50 min. The slides were then developed in freshly prepared Cy3-tyramide chromogenic reagent (Roche) in the dark for 5 min at room temperature to mark the tissue and then washed three times in PBS for 10 min each wash. The slides were incubated with DAPI (Servicebio) for 8 min in the dark to stain the nuclei. Finally, the presence of TIBOV RNA was examined using a positive fluorescence microscope (Nikon, Tokyo, Japan).

### 2.11. Statistical Analysis

Statistical analyses were performed using GraphPad Prism 8 software (GraphPad Software Inc., San Diego, CA, USA). Each sample was tested in triplicate, and all values are presented as the mean ± standard deviation. Differences between groups were analyzed using multiple *t*-test, and a probability (*p*-value < 0.05) was considered statistically significant.

## 3. Results

### 3.1. Meta-Virome Sequencing Data

From the samples collected on 16 October 2023, a total of 222,471,178 raw reads were acquired. After the removal of contaminating host sequences, adapter sequences, and other low-quality sequences, the total effective number of reads was 25,576,604 (11.50%). Subsequently, the eukaryote reads, bacterial sequences, and archaea sequences were eliminated, and the virus reads were 194,938 (45.63%), 480,154 (1.95%), and 186,300 (38.84%) in samples ML10-1, ML10-2, and ML10-3, respectively (Table 1). In the samples collected on 15 December 2023, a total of 243,429,804 raw reads were acquired. After the removal of the contaminating host sequences, adapter sequences, and other low-quality sequences, the total effective number of reads was 7,159,086 (2.94%). Subsequently, the eukaryote reads, bacterial sequences, and archaea sequences were eliminated, and the numbers of virus reads were 392,246 (40.59%), 72,922 (1.35%), and 163,474 (20.50%) in samples ML12-1, ML12-2, and ML12-3, respectively (Table 1). There were 13 and 15 viral families in the samples collected on October 16, 2023, and December 15, 2023, respectively (data not shown).

### 3.2. Homology and Phylogeny Analysis of the TIBOV Sequence

**Figure 1:**
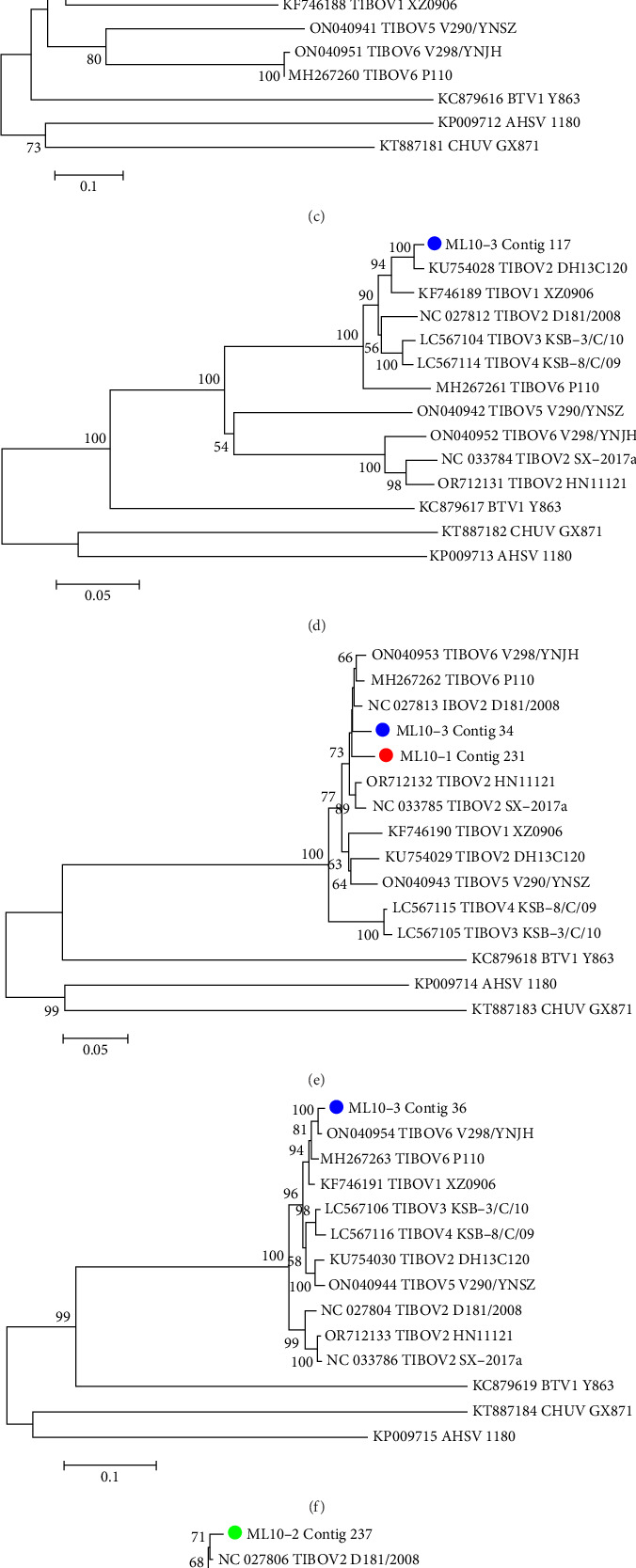
Phylogenetic analysis based on the partial nucleotide sequence of TIBOV genes detected in the samples and reference TIBOV strains from GenBank. The trees of VP1 (A), VP2 (B, C), VP3 (D), VP4 (E), NS2 (F), NS1 (G), and VP6 (H) gene were constructed by the neighbor-joining method in MEGA 6.0 and tested by bootstrapping 1000 replicates. TIBOV sequences in the sample ML10-1 were labeled with red dot (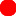), sequence in the sample ML10-2 was labeled with green dot (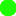) and sequences in the sample ML10-3 were labeled with blue dots (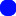). The sequences of reference TIBOV strains are indicated as GenBank accession number TIBOV serotype and strain name. Sequences of homologous gene of BTV1 Y863, CHUV GX871, AHSV 1180 strains are seted as outlier virus group to root the the phylogenetic trees.

TIBOV sequences were detected in all three samples collected on October 16, 2023 (Table 1). However, none were detected in the samples collected on December 15, 2023. Specifically, four contigs (*VP1*, *VP2*, *VP4*, and *VP6*), one contig (*NS2*), and 11 contigs (one contig of *VP3*, *VP6*, *NS1*, and *NS2*; two contigs of *VP4* and *VP6*; three contigs of *VP1*) were identified as TIOBV genes in samples ML10-1, ML10-2, and ML10-3, respectively. The length of these contigs ranged from 500 to 1263 bp, and they shared high nucleotide identity (95.01%–99.70%) with the respective segments of referenced TIBOV strains (Table 2). Contig ML10-1-297 (*VP2* gene) showed the highest sequence identity with TIBOV-1 strain XZ0906 (GenBank accession: KF746188.1), indicating that the TIOBV strain in sample ML-10-1 belonged to the TIBOV-1 serotype. Contigs ML10-3-57 and ML10-3-208 showed the highest identity with the TIBOV-2 strains SX-2017a (GenBank accession: KX455488.1) and HN11121 (GenBank accession: OR712130.1), respectively, indicating that the TIOBV strains in sample ML-10-3 were TIBOV-2 serotypes. Phylogenetic analysis showed that the TIBOV sequences in the samples did not cluster (Figure 1), indicating that the TIBOV strains in the different samples were closely related to the different TIBOV reference strains.

### 3.3. Clinical Symptoms

**Figure 2:**
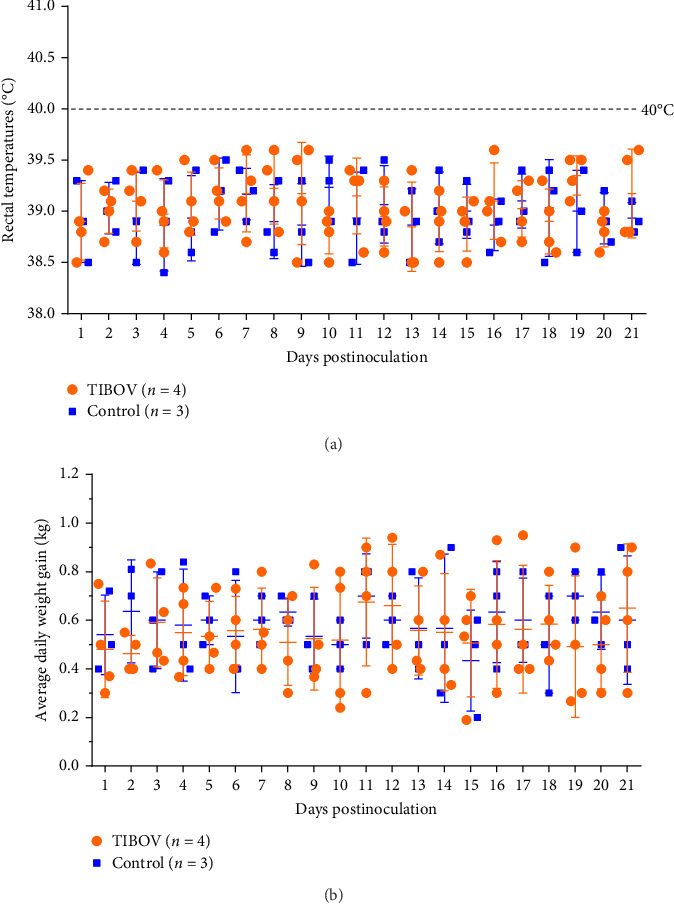
The rectal temperature and weight gain of TIBOV/V290/YNSZ-infected piglets and mock-infected controls. (A) Rectal temperatures of piglets infected with TIBOV/V290/YNSZ and mock-infected, mean ± SD temperature (°C) was shown, and the fever cut off value was set at 40.0°C. (B) Average daily weight gain of the piglets inoculated with TIBOV/V290/YNSZ and mock-infected, mean ± SD weight gain (kg) were shown.

**Figure 3:**
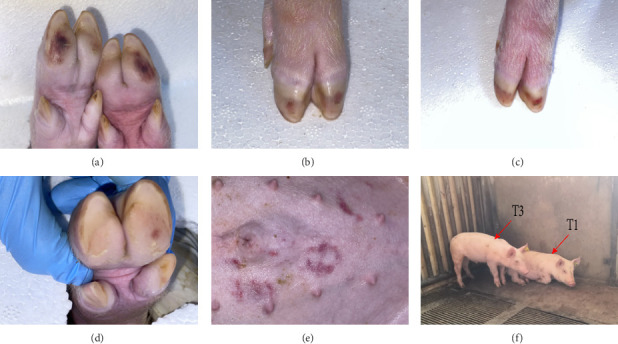
The clinical symptoms of TIBOV/V290/YNSZ infected piglets. The hemorrhage nodes at hooves of infected pigs (A–D). The hemorrhage nods on abdominal skin of TIBOV/V290/YNSZ infected pigs T1 (E). TIBOV/V290/YNSZ infected pig T1 decreased activity and favored lying down (F).

To assess the pathogenicity of TIBOV in pigs, four piglets (31 days old) were intravenously inoculated with TIBOV strain V290/YNSZ. None of the challenged pigs died during the study period, and clinical symptoms such as fever (Figure 2A), respiratory symptoms, anorexia (Figure 2B), and diarrhea were not observed. Hemorrhage nodes were observed in the hooves of infected pigs from 7 to 11 dpi and on the abdominal skin of one pig at the same time but disappeared from 13 to 16 dpi (Figure 3A–E). In addition, pig T1 showed decreased activity and favored lying down from 7 to 9 dpi (Figure 3F). The appetite of this pig was not affected, and the mobility was gradually on the mend from 12 dpi. These data suggested that TIBOV is mildly pathogenic in piglets.

### 3.4. Viremia Analysis and Viral Load Detection in Tissues

**Figure 4:**
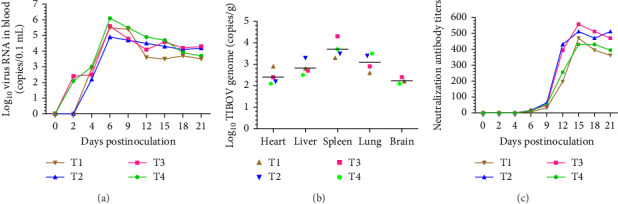
Viremia, virus load and the serum neutralization antibody titers detection. (A) Detection of duration of viremia of the piglets challenged with TIBOV/V290/YNSZ, and viremia persisted for more than 21 days post-TIBOV-infection. (B) Viral load detection in tissues and TIBOV can be detected in heart, liver, spleen, lung, and brain. (C) nAbs against TIBOV/V290/YNSZ of challenged piglets, the neutralization titer of serum was defined as the highest serum dilution giving 50% of virus growth was inhibited. T1 to T4 represented four pigs in the infected group.

To monitor the duration of viremia post-TIBOV-infection in piglets, the nucleic acid of TIBOV strain V290/YNSZ was detected in blood samples using the TIBOV pan-RT-qPCR method developed in our previous study [4]. TIBOV RNA was detected in the blood of two inoculated pigs (T3 and T4) at 2 dpi, with titers of 10^2.1^ and 10^2.4^ copies/μL, respectively (Figure 4A), while the blood samples of the other two pigs in the experimental group became positive at 4 dpi. The viral load in the blood of all animals peaked at 6 dpi, with the virus titer ranging from 10^4.9^ to 10^6.1^ copies/μL (Figure 4A), thereafter the viral load remained at high levels (10^3.5^ to 10^4.3^ copies/μL) until the end of the experiment (Figure 4A). Therefore, in this study, TIBOV viremia lasted until 21 dpi in pigs (Figure 4A). The tissue viral load detection results showed that V290/YNSZ replicated to the highest titer (10^4.1^ copies/g) in the spleen (Figure 4B), and the viral load decreased gradually in other tissues. The mean viral loads were 10^2.4^ copies/g, 10^2.9^ copies/g, 10^3.1^ copies/g, and 10^2.2^ copies/g in the heart, liver, lung, and brain, respectively (Figure 4B). Viral RNA was not detected before inoculation or in mock-infected piglets (data not shown).

### 3.5. nAb Levels

nAbs against V290/YNSZ in the serum of infected piglets were detected using SNT. All TIBOV-infected piglets seroconverted and generated nAb at 6 dpi, with a mean titer of 1:12.75, then gradually increased, reaching the highest levels around 15 dpi, with a mean titer of 1:431 to 1:558 (Figure 4C). The nAbs remained constant at about 1:362 to 1:512 until the end of the study period (Figure 4C). No V290/YNSZ-specific antibodies were detected in sera collected from mock-infected animals throughout the study (data not shown).

### 3.6. Gross Pathology and Histopathological Analysis

**Figure 5:**
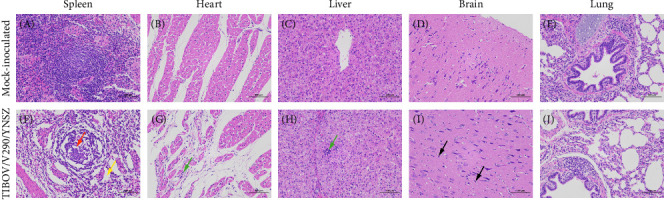
Histopathological analysis of the piglets challenged with TIBOV/V290/YNSZ through hematoxylin and eosin (H&E)-staining. Comparing with the organs of the mock-inoculated piglets (A–E), the white pulp of the spleen of the challenged pig was atrophied, germinal center was not clear and the lymphocytes decreased (red arrow), and the medullary cord was incompact and the septa widened (yellow arrow) (F). Local lymphocytic infiltration in the heart and liver (green arrow) (G,H), and mild glial cell hyperplasia in the brain of the challenged pigs (black arrow) (I), and any pathological lesion was not observed in the lung (J).

At 21 dpi, the pigs were euthanized and necropsied; however, no typical gross lesions were observed. To assess pathological injury to the organs, the spleen, heart, liver, lungs, and brain were subjected to histopathological examination. Compared to the control group, the white pulp of the challenged pigs was atrophied, the germinal center was not clear, the number of lymphocytes decreased (red arrow), the medullary cord was incompact, and the septa widened (yellow arrow) (Figure 5E). Local lymphocytic infiltration in the heart and liver (green arrow) (Figure 5G,H), and mild glial cell hyperplasia were observed in the brains of challenged pigs (black arrow) (Figure 5I), but no obvious pathological lesions were observed in the lungs (Figure 5J).

**Figure 6:**
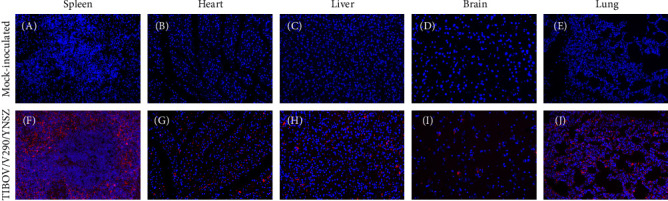
Detect the distribution of TIBOV viral nucleic acids in the spleen, heart, liver, brain and lung of TIBOV/V290/YNSZ infected piglets through Cy3-tyramide signal amplification fluorescence in situ hybridization (TSA-FISH). Any positive signals (red fluorescence) were not observed in the organs of mock-inoculated piglets (A–E). Cy3 signal (red fluorescence) was detected in all organs (F–J), and the strongest signal (red fluorescence) was detected in the white pulp of spleen (F), and the cell nucleus was stained blue with DAPI.

Spleen, liver, heart, lung, and brain slices were subjected to TSA-FISH to determine the distribution of viral nucleic acids in the organs of the infected animals. A specific signal could be detected in all tested tissues (Figure 6F–J). The strongest signal was observed in the red pulp of the spleen (Figure 6F), and the weakest signal was observed in the brain (Figure 6J). Additionally, no specific signal was observed in the organs of uninoculated piglets (Figure 6A–E). Collectively, the TIBOV strain V290/YNSZ could proliferate in several organs and cause lesions in infected pigs; thus, we inferred that TIBOV is a potential pathogen in pigs.

### 3.7. Cytokine Gene mRNA Levels in Peripheral Blood Mononuclear Cells (PBMCs) and Concentrations in the Serum of TIBOV-Infected Piglets

**Figure 7:**
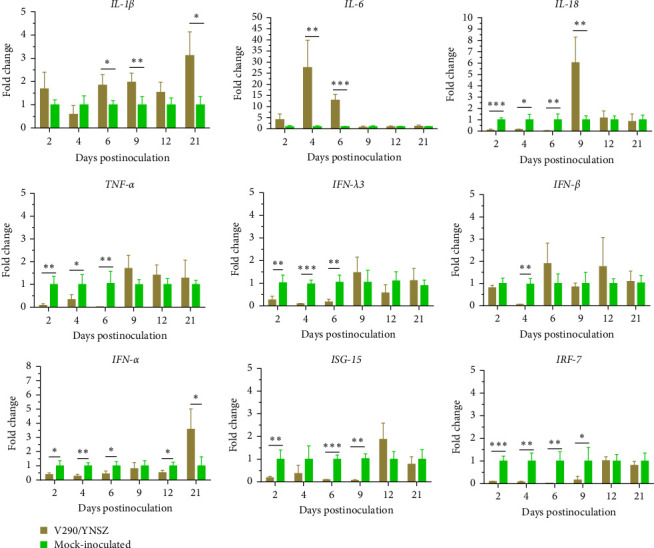
The expression profiles of multiple immune-related cytokine genes (*IL-1*β, *IL-6*, *IL-18*, *TNF-α*, *IFN-α*, *IFN-β*, *IFN-λ3*, *ISG-15*, and *IRF-7*) in the PBMCs of the TIBOV/V290/YNSZ inoculated piglets at 2, 4, 6, 9, 12, and 21 dpi. The relative expression levels of the target genes were normalized to the *β*-actin gene and calculated by the delta–delta cycle to threshold (2^−ΔΔCt^) method. The *Y*-axis represents the fold change of target gene expression in the experimental group versus that of the control group. Data are expressed as means ± SD. The significance of differences was analyzed with multiple *t*-test. *⁣*^*∗*^*p*  < 0.05, *⁣*^*∗∗*^*p*  < 0.01, *⁣*^*∗∗∗*^*p*  < 0.001.

The mRNA levels of multiple cytokines were determined in the PBMCs of piglets infected with TIBOV and mock groups using RT-qPCR. As shown in Figure 7, the transcription level of *IL-1*β was up-regulated significantly at 6, 9, and 21 dpi; *IL-6* levels were enhanced significantly at 4 dpi (29.60-fold) and 6 dpi (12.94-fold) (*p* < 0.001). However, *IL-18* and *TNF-α* levels were significantly reduced at 2, 4, and 6 dpi, and the transcription levels ranged from 2.67% (*IL-18*, 6 dpi) to 33.53% (*TNF*-*α*, 4 dpi) relative to the control group. At 9 dpi, *IL-18* was significantly up-regulated (*p* < 0.01) and then reduced to normal levels (Figure 7). *INF-λ3* levels were reduced significantly (*p* < 0.01) at 2, 4, and 6 dpi (Figure 7), and *IFN-α* levels were reduced significantly (*p* < 0.05) at 2, 4, 6, and 12 dpi, and were upregulated significantly (*p* < 0.05) at 21 dpi (Figure 7), while *IFN-β* was reduced significantly (*p* < 0.01) at 4 dpi and maintained at relatively constant levels at all other time points (Figure 7). Additionally, the mRNA levels of *ISG-15* decreased significantly (*p* < 0.01) at 2, 6, and 9 dpi, and *IRF-7* levels decreased significantly (*p* < 0.05) from 2 to 9 dpi compared to the levels in the mock-infected group (Figure 7).

**Figure 8:**
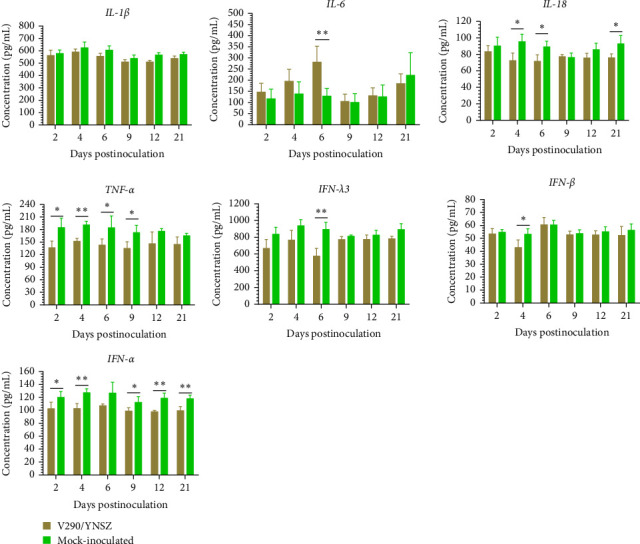
The protein concentrations of the *IL-1β*, *IL-6*, *IL*-*18*, *TNF*-*α*, *IFN*-*α*, *IFN*-*β* and *IFN*-*λ3* in serum of the piglets challenged by TIBOV/V290/YNSZ. Data are expressed as means ± SD. The significance of differences was analyzed with multiple *t* test. *⁣*^*∗*^*p*  < 0.05, *⁣*^*∗∗*^*p*  < 0.01.

The concentrations of porcine cytokines, including *IL-1*β, *IL-6*, *IL-18*, *TNF-α*, *IFN-α*, *IFN-β*, and *IFN-λ3*, were determined using enzyme-linked immunosorbent assays, and the results were consistent with the mRNA levels. In general, the concentrations of *IL-18*, *TNF-α*, *IFN-λ3*, *IFN-α*, and *IFN-β* were lower in infected piglets than that in mock-inoculated piglets (Figure 8), and the concentration of *IL-6* was elevated compared to the control group, while *IL-1*β did not change significantly. Specifically, the concentration of *IL-18* in the TIBOV-challenged piglets decreased significantly at 4, 6, and 21 dpi (*p* < 0.05, Figure 8), and the *TNF*-*α* concentration decreased significantly at 2, 4, 6, and 9 dpi (*p* < 0.05, Figure 8), and the *IFN*-β and *IFN*-*λ3* concentrations decreased significantly at 4 dpi (*p* < 0.05, Figure 8) and 6 dpi (*p* < 0.01, Figure 8) respectively. The *IFN*-*α* concentration decreased significantly from 2 to 21 dpi (*p* < 0.05, Figure 8), except at 6 dpi.

## 4. Discussion

Traditional etiological detection methods target specific pathogens, and some unknown or novel pathogens cannot be detected efficiently. Culture-independent viral metagenomic sequencing overcomes the limitation of traditional methods of virus identification and provides a great opportunity for large-scale detection of known and unknown viruses present in various samples [29]. Numerous novel viruses have recently been discovered in humans [30], animals [31], plants [32], and insects [33] using this technology. In previous studies, specific antibodies [8] and viral nucleic acids [4] of TIBOV have been detected in blood samples collected from pigs; however, no TIOBV sequence was obtained from these samples; and thus, the molecular epidemiology of TIOBV in pigs remains elusive. In the present study, 16 contigs, including seven segments (VP1–VP4, VP6, NS1, and NS2), were obtained from porcine tissues through viral metagenomic sequencing, providing new evidence that TIBOV can infect pigs and making it possible to analyze the genetic evolution characteristics of the TIBOV strains prevalent in pigs. According to the results of our study, TIOBV-infected pigs showed high identity with strains isolated from *Culicoides*, mosquitoes, and cattle, clustered together with other isolates, with no separate branches observed.

Several viruses of the genus *Orbivirus* have been confirmed to be pathogenic to animals, such as BTV, which infects domestic and wild ruminants and leads to bluetongue disease in some breeds of sheep, with a mortality rate of 5%–30% [34]. EHDV can cause severe hemorrhagic symptoms in white-tailed deer, with mortality rates as high as 90% [35]. African horse sickness virus can infect equids (horses, mules, donkeys, and zebras), and the acute disease in horses and zebras (the lung form) shows a high mortality rate (80%–90%) [36]. In previous studies, several researchers have reported that TIBOV is lethal to suckling mice [4, 8], while the pathogenicity of TIBOV to pigs is completely unknown. Therefore, we conducted artificial infections of 31-day-old piglets with TIOBV to access the pathogenicity of the virus to pigs. TIBOV showed mild pathogenicity in piglets, and only slight hemorrhages on the hooves (four piglets) and abdomen (one piglet) were observed after TIOBV challenge. These hemorrhagic symptoms were similar to those of BTV and EHDV infections [34, 35]; however, facial edema, oral erosions, and ulcers were not observed. It is noteworthy that no overt lesions were found in the spleen during autopsy; however, conspicuous histopathological lesions were confirmed by hematoxylin and eosin staining. We speculated that since the autopsy was conducted at 21 dpi, the damage to the spleen induced by TIBOV infection was partially repaired, and obvious gross lesions had already regressed; however, it would take more time to repair the histological lesions, and some histological lesions might be irreversible.

One of the critical nodes of the arbovirus transmission cycle is the establishment of sufficiently high- and long-term viremia in vertebrate hosts, because prolonged viremia offers a long window phase for the transmission of arboviruses by hematophagous arthropod vector biting [37–39]. In our study, TIBOV proliferated rapidly in the blood of piglets after infection, and viral RNA was detectable in the blood at 2 dpi (10^2.1^ and 10^2.4^ copies/μL), and peaked at 6 dpi (10^4.9^ to 10^6.1^ copies/μL), while the virus load remained at high levels (10^3.5^ to 10^4.3^ copies/μL) at 21 dpi. The viremia kinetics reported here are similar to those of other orbiviruses, such as BTV and EHDV, for which long-term viremia has previously been reported in sheep and cattle [40, 41]. The peak period of viremia lasts for a relatively short time (less than 1 week) postinfection with Japanese encephalitis virus and Seneca Valley virus [42–44], both of which are important arbovirus pathogens in pigs. TIBOV is not highly pathogenic to pigs; however, infected pigs may act as long-term reservoirs because of the long-lasting, high viremia postinfection, making it possible to establish sustained epidemic cycles of the virus in nature.

The results of viral load detection showed that viral nucleic acid could be detected in several organs, demonstrating that TIBOV had broad tissue tropism, and the virus could proliferate in multiple tissues. Notably, the highest viral load (10^4.1^ copies/g) was observed in the spleen; the strongest signal was observed in the spleen using TSA-FISH, and the most serious histopathological lesions were found in the spleen. Taking these results together, we speculate that the spleen is the most important target organ of TIBOV. It is well known that the spleen is an important immune organ in animals and that the immune system might be affected by a TIOBV-infected spleen. Serious histopathological lesions can be found in the spleens of pigs after infection with immunosuppressive pathogens, such as CSFV [45], PRRSV [46], and PCV-2 [47], and subsequent infection by opportunistic pathogens might ensue [48–50], causing the disease to worsen. Therefore, the impact of TIBOV infection on the immune system of pigs and subsequent infection with other pathogens should not be ignored, although TIBOV did not cause direct or serious damage to the infected pigs.

dsRNA is an important pathogen-associated molecular pattern for the host innate immune system. It can be sensed by retinoic acid-inducible gene-I and melanoma differentiation-associated factor 5 in the cytoplasm, which induces the expression of *IFNs* and elicits the production of pro-inflammatory cytokines in vivo, ultimately promoting an antiviral innate immune response in host cells to limit viral infection [51, 52]. In contrast, some single-stranded RNA viruses (Ebola virus, influenza A virus, and hepatitis C virus) [53–55] and dsRNA viruses (BTV and reovirus) [56, 57] are able to escape the host immune response using different strategies, and immune evasion is critical for prolonged viral infection. In our study, the transcription levels of *TNF-α*, *IL-18*, *IFN-α*, and *IFN-λ3* were reduced in the early stage of virus infection (within 6 dpi), the transcription levels increased gradually but were maintained at a level equivalent to that of the control group from dpi 12 onward. Unexpectedly, the serum concentration of *IFN-α* was significantly lower in the infected group than the control group from 2 dpi to 21 dpi. We anticipated that the production of *IFNs* would be impaired by TIBOV infection in pigs, and an effective antiviral immune response would not be established post-infection. The transcription level of *IRF-7* continued to decrease after infection, and we hypothesized that the transcription of *IRF-7* might be inhibited by TIBOV, eventually reducing the production of *IFNs*. However, the significant increase of the transcriptional levels of IL-6 was detected in the early stage of TIBOV infection (2, 4, and 6 dpi). And it is a generally acknowledged fact that IL-6 is a critical prominent proinflammatory cytokine released during infection or tissue injury that contributes to both innate and adaptive immune responses [58]. The increase of the levels of IL-6 indicated that TIBOV infection has induced the inflammatory response in piglets. To further explore the effects of TIBOV infection on the host immune system, in future studies, we will detect variations in different PBMC populations produced by TIBOV infection, sequence the transcriptome of the PBMCs, and study the interactions between TIBOV and the animal's immune system.

## 5. Conclusions

TIBOV strains prevalent in pigs are closely related to those isolated from other hosts. TIBOV elicited long-term viremia with mild clinical symptoms in piglets. The spleen was the target organ of TIBOV proliferation, and the host immune response may be inhibited slightly in the early stage of virus infection.

## Figures and Tables

**Table 1:** Sample collection information and generated data through viral metagenomic sequencing.

Sample name	Location	Longitude	Latitude	Collection Date	Numbers	Total reads number	Viral reads	Viral contigs	TIBOV contigs
ML10-1	Peng Pu	E 103°22'12”	N 23°57'47”	16 October 2023	3	72,283,652	194,938	141	4
ML10-2	Xin Shao	E 103°27'34”	N 24°15'11”		4	73,586,518	480,154	107	1
ML10-3	Mi Yang	E 103°27'01”	N 24°25'33”		4	76,601,008	186,300	124	11

ML12-1	Hong Xi	E 103°19'41”	N 24°09'40”	15 December 2023	3	82,194,584	966,418	150	0
ML12-2	Mi Yang	E 103°27'02”	N 24°25'48”		3	78,371,964	5,395,102	197	0
ML12-3	Peng Pu	E 103°22'11”	N 23°58'12”'		4	82,863,256	797,566	195	0

**Table 2:** Information of contigs of TIBOV in the samples.

Sample	Contigs	Length (bp)	No. of reads	Matched strains (Genbank no.)	Query cover	Identity (%)	*E*-value	Covered range	Covered viral gene
ML10-1	ML10-1-70	1263	48	YN12246(KP099640.1)	100	95.01	1.40E-295	1680–2942	VP1
	ML10-1-297	562	28	XZ0906(KF746188.1)	100	97.51	1.00E-121	542–1103	VP2
	ML10-1-231	642	34	D181/2008(NC_027813.1)	98.75	98.26	1.70E-148	574–1207	VP4
	ML10-1-217	659	66	YN15-283-01(MT793644.1)	99.7	98.33	2.20E-96	87–743	VP6

ML10-2	ML10-2-237	565	14	YN15-283-01(MT793643.1)	99.65	98.58	5.5E-125	174–736	NS2

ML10-3	ML10-3-271	500	50	YN12246(KP099640.1)	100	98.20	6.30E-97	3210–3709	VP1
	ML10-3-243	523	24	YN12246(KP099640.1)	100	97.32	5.00E-110	2319–2841	VP1
	ML10-3-95	781	141	YN12246(KP099640.1)	96.29	98.67	0	1327–2078	VP1
	ML10-3-208	568	68	SX-2017a(KX455488.1)	98.06	98.05	0	211–672	VP2
	ML10-3-57	974	112	HN11121(OR712130.1)	98.46	98.66	4.70E-224	1196–2166	VP2
	ML10-3-117	726	72	XZ0923(OR712121.1)	98.76	99.58	1.10E-167	715–1431	VP3
	ML10-3-34	1154	317	V298/YNJH(ON040953.1)	100	99.70	2.60E-281	60–1213	VP4
	ML10-3-203	571	250	D181/2008(NC_027813.1)	100	98.97	0	1473–1958	VP4
	ML10-3-100	768	211	V290/YNSZ(ON040948.1)	98.57	99.34	2.30E-118	203–959	VP6
	ML10-3-122	709	170	YNV/17-14(ON211606.1)	99.86	99.44	1.70E-153	171–878	NS2
	ML10-3-36	1131	72	V298/YNJH(ON040954.1)	100	98.85	1.80E-283	202–1332	NS1

## Data Availability

All data of this study are available from the corresponding author upon request.
