# Supplementary material for: Metavirome Identification and Pathogenicity Evaluation of Tibet Orbivirus in Pigs
Source: Transbound Emerg Dis. 2025 Nov 17;2025:6628384. doi: 10.1155/tbed/6628384 (PMC12643696; doi:10.1155/tbed/6628384)
Supplement: Supporting Information — Additional supporting information can be found online in the Supporting Information section. Table S1: The RT-qPCR primers used to measure mRNA levels of various cytokines. [file 6628384.f1.docx]

**TABLE S1.** The RT-qPCR primers used to measure mRNA levels of various cytokines.

| **Primer Name** | **Sequence (5'→3')** | **Reference** |
| --- | --- | --- |
| pISG15-F | GATCGGTGTGCCTGCCTTC | [25] |
| pISG15-R | CGTTGCTGCGACCCTTGT |  |
| pIRF7-F | CTGCGATGGCTGGATGAA | [26] |
| pIRF7-R | TAAAGATGCGCGAGTCGGA |  |
| pIFN-β-F | AGCAGATCTTCGGCATTCTC | [24] |
| pIFN-β-R | GTCATCCATCTGCCCATCAA |  |
| pIFN-λ3-F | GTTCAAGTCTCTGTCCCCAC |  |
| pIFN-λ3-R | GCTGCAGTTCCAGTCCTC |  |
| βActin−F | CCCAGCACCATGAAGATCAA | [23] |
| βActin−R | GATCCACATCTGCTGGAAGG |  |
| IFN-α-F | TTCTGCACTGGACTGGATC |  |
| IFN-α-R | TCTGTGGAAGTATTTCCTCACAG |  |
| IL-1β-F | ACCCAAAACCTGGACCTTGG | [21] |
| IL-1β-R | CATCACAGAAGGCCTGGGAG |  |
| IL-6-F | CTCATTAAGTACATCCTCGG |  |
| IL-6-R | GTCTCCTGATTGAACCCAGA |  |
| pTNF-a-F | CCTACTGCACTTCGAGGTTATC |  |
| pTNF-a-R | ACGGGCTTATCTGAGGTTTG |  |
| IL-18-F | CGTGTTTGAGGATATGCCTGATT | [22] |
| IL-18-R | TGGTTACTGCCAGACCTCTAGTGA |  |

[21] Zhou P., Li L. F., Zhang K., et al. Deletion of the H240R Gene of African Swine Fever Virus Decreases Infectious Progeny Virus Production Due to Aberrant Virion Morphogenesis and Enhances Inflammatory Cytokine Expression in Porcine Macrophages. *Journal of Virology.* (2022). *96*, no., e0166721. doi:10.1128/JVI.01667-21.

[22] Razzuoli E., Mignone G., Lazzara F., et al. Impact of cadmium exposure on swine enterocytes. *Toxicology Letters.* (2018). *287*, no., 92-99. doi:10.1016/j.toxlet.2018.02.005.

[23] Temeeyasen G., Sinha A., Gimenez-Lirola L. G., et al. Differential gene modulation of pattern-recognition receptor TLR and RIG-I-like and downstream mediators on intestinal mucosa of pigs infected with PEDV non S-INDEL and PEDV S-INDEL strains. *Virology.* (2018). *517*, no., 188-198. doi:10.1016/j.virol.2017.11.024.

[24] Deng X., van Geelen A., Buckley A. C., et al. Coronavirus Endoribonuclease Activity in Porcine Epidemic Diarrhea Virus Suppresses Type I and Type III Interferon Responses. *Journal of Virology.* (2019). *93*. doi:10.1128/JVI.02000-18.

[25] Yang K., Xue Y., Niu H., et al. African swine fever virus MGF360-11L negatively regulates cGAS-STING-mediated inhibition of type I interferon production. *Veterinary Research.* (2022). *53*, no., 7. doi:10.1186/s13567-022-01025-0.

[26] Xue Q., Liu H., Zhu Z., et al. Seneca Valley Virus 3C(pro) abrogates the IRF3- and IRF7-mediated innate immune response by degrading IRF3 and IRF7. *Virology.* (2018). *518*, no., 1-7. doi:10.1016/j.virol.2018.01.028.
